# Formulation and Optimization of Sustained Release Tablets of Venlafaxine Resinates Using Response Surface Methodology

**DOI:** 10.4103/0250-474X.57287

**Published:** 2009

**Authors:** Ashwini R. Madgulkar, M. R. Bhalekar, V. J. Kolhe, Y. D. Kenjale

**Affiliations:** AISSMS College of Pharmacy, Kennedy Road, Near RTO, Pune-411 001, India

**Keywords:** Venlafaxine hydrochloride, ion exchange resin, sustained release, matrix, factorial design, optimization, response surface methodology

## Abstract

The aim of the current study was to design sustained release matrix tablets of venlafaxine hydrochloride using ion exchange resin with the incorporation of hydrophilic and hydrophobic polymer combinations. Venlafaxine HCl was loaded onto Indion 244 by batch method and then resinate were wet granulated with ethyl cellulose and blended with hydroxypropylmethylcellulose and compressed. A central composite design for 2 factors at 3 levels each was employed to systematically optimize drug release profile at 2 h and at 18 h. Hydroxypropylmethylcellulose and ethylcellulose were taken as the independent variables. Response surface plots and contour plots were drawn, and optimum formulations were selected by feasibility and grid searches. Resinate shows inadequate sustained release profile. Compressed matrices exhibited the anomalous release mechanism, as the value of release rate exponent (*n*) varied between 08109 and 08719, resulting in regulated and complete release until 20 h. Validation of optimization study, performed using five confirmatory runs, indicated very high degree of prognostic ability of response surface methodology, with mean percentage error as 1.152±1.88%. Regulated drug release study indicates that the hydrophilic and hydrophobic matrix tablets of venlafaxine resinate prepared using hydroxypropylmethylcellulose and ethylcellulose, can successfully be employed as a once-a-day oral controlled release drug delivery system.

Venlafaxine is a unique antidepressant that differs structurally from other currently available antidepressant[1]. Venlafaxine and its active metabolite, o-desmethylvenlafaxine, inhibit the neuronal uptake of norepinephrine, serotonin and to a lesser extent dopamine[2,3], but have no monoamine oxidase inhibitory activity and a low affinity for brain muscarinic, cholinergic, histaminergic or alpha adrenergic receptors[4,5]. Hence, it lacks the adverse anticholinergic, sedative and cardiovascular effects of tricyclic antidepressants. The steady state half lives of venlafaxine and ODV are 5 h and 11 h, respectively, necessitating the administration, 2 or 3 times daily so as to maintain adequate plasma levels of drug[6].

The present research was directed towards the development of sustained release dosage form of venlafaxine HCl using ion exchange resin with incorporation of polymer matrix Indion 244 IER and different polymers such as hydroxylpropylmethylcellulose (HPMC) K 15 M and ethylcellulose (EC) 7 cps were used. Indion 244 is a strong cation exchange resin with SO_3_^−^ H^+^ functionality which exchanges cations stoichiometrically in an equilibrium driven reaction. Due to the presence of SO_3_^−^ H^+^ group Indion 244 shows ionization at all body pH values. However simple drug-resin complexes may not satisfy the requirement of sustained release, in such cases resinates are incorporated into the matrix systems, microencapsulated or coated.

HPMC is mixed alkyl hydroxypropylcellulose ether containing methoxyl and hydroxypropyl groups. The hydration rate of HPMC depends on the nature of these substituents HPMC-tablets hydrate upon contact with water and a rate-controlling gel layer forms around a solid inner core. A rapid formation of the gel layer is a prerequisite for the retardation of the drug release; otherwise, hydrophilic drugs would be released rapidly[7].

Response surface methodology (RSM) is a widely practiced approach in the development and optimization of drug delivery devices[8–12]. Based on the principle of design of experiments (DOE), the methodology encompasses the use of various types of experimental designs, generation of polynomial equations, and mapping of the response over the experimental domain to determine the optimum formulation(s)[10–12]. The technique requires minimum experimentation and time, thus proving to be far more effective and cost-effective than the conventional methods of formulating dosage forms. The tablets incorporating resinate into HPMC matrix were developed employing 3^2^ factorial design, employing concentration of ethyl cellulose 7 cps and HPMC K15M as factors, release at 2 h and release at 18 h as response.

## MATERIAL AND METHODS

Venlafaxine HCl was obtained from Lupin Research Park, Pune. Indion 244 was provided by Ion Exchange India, Mumbai. HPMC K15M and EC 7cps were obtained as gift samples from M/s Colorcon Asia Pvt Ltd, Mumbai, India.

### Preparation and evaluation of venlafaxine HCl-resin complex (DRC):

Resinate was prepared by batch process[13]. An accurately weighed amount of venlafaxine HCl (1 g) was taken and dissolved in 100 ml of deionised water. A known weight of Indion 244 was added to the same and was stirred on a magnetic stirrer. Time to reach equilibrium was determined by periodically measuring concentration of the drug in solution spectrophotometrically. Resinate thus formed was filtered and washed with deionised water. It was then dried at 50°. The amount of drug loaded on the resin was obtained by subtracting the remaining amount of drug in the final filtrate from initial amount. FT-IR studies were carried out on drug, resin and resinate to determine the formation of complex of drug and resin.

### *In vitro* drug release studies:

DRC of drug was subjected to *in vitro* dissolution studies in phosphate buffer (pH 7.2, 900 ml, 37°±0.5°) using USP dissolution apparatus type 2 (paddle type, 100 rpm). An accurately weighed (75 mg drug equivalent) DRC were taken in dissolution medium. Dissolution studies were carried out using conditions mentioned above. At regular time interval aliquots of dissolution medium (10 ml) were taken, filtered and absorbance was measured by UV spectroscopy. The medium was replaced with equal volume of fresh dissolution fluid.

### Effect of pH on drug release from DRC:

A claimed advantage of ion exchange delivery system is that release of drug is independent of pH of the dissolution medium. This prospect was investigated by preparing buffer solutions of different pH (1.2, and 7.2) with ionic strength adjusted to μ ≃ 0.2 using NaCl. *In vitro* release drug resinate was carried out at these pH, using conditions described earlier.

### Formulation of matrix tablets using 3^2^ factorial design:

Composition of different formulations as per Table 1 prepared using varying amounts of the polymers. The preparation process involved two steps first DRC were wet granulated with EC using PVP K30 in isopropyl alcohol as a granulating agent. The granules were dried at 60° and pass the granules through 30#. Second step involves blending of granules with HPMC and dicalcium phosphate. Then granules were lubricated with magnesium stearate. The tablets were compressed into flat-faced punches of 8 mm diameter using Rimek Mini Press-II MT tablet compression machine.

**TABLE 1:** COMPOSITION OF VENLAFAXINE HCL SUSTAINED RELEASE HYDROPHILIC AND HYDROPHOBIC COMPRESSED MATRICES

Ingredients	Quantity (mg)
DRC	Equivalent to 75 mg of drug
Ethylcellulose 7 cps	41.62-124.86
Hydroxypropylmethylcellulose K 15M	41.62-124.86
Magnessium stearate	4.5
Di calcium phosphate	q.s. to make 450s

*DRC= drug-resin complex, q.s.= quantity sufficient.

### Experimental design:

A 3-level full factorial design comprising 9 full factorial design points was applied as shown in Table 2. This design generally involves dependent variables Y and independent or controlled variables X_1_, X_2_. The two independent formulation variables selected for this study were X_1_, HPMC K 15 M; X_2_, EC 7 cps. The levels of independent variables are shown in Table 2. The dependent variables were Y_1_,% of drug released at 2 h; Y_2_, % of drug released release at 18 h.

**TABLE 2:** 3^2^ FULL FACTORIAL EXPERIMENTAL DESIGN LAYOUT

Trial No.	Coded Factor Levels	
	
	X_1_	X_2_
F_1_	-1	-1
F_2_	-1	0
F_3_	-1	1
F_4_	0	-1
F_5_	0	0
F_6_	0	1
F_7_	1	-1
F_8_	1	0
F_9_	1	1

F_1_-F_9_: Codes for 9 formulations designed by factorial design, Coded levels for X_1_: HPMC in mg -1 is 41.62, 0 is 83.24 and 1 is 124.86 and for X_2_: Ethyl cellulose in mg are -1 is 41.62, 0 is 83.24 and 1 is 124.86.

### Tablet assay and physical evaluation:

Drug content of all the batches was determined. For this purpose ten tablets were weighed and crushed in a small glass mortar with pestle. The fine powder was weighed to get 100 mg equivalent of venlafaxine HCl, and transferred to 250 ml conical flask containing 100 ml of 1N HCl and stirred for 4 h on magnetic stirrer. Dispersion was filtered and the filtrates obtained were analyzed spectrophotometrically. Tablets were also evaluated for uniformity of weight and thickness. Tablets were examined for friability using a Roche type friabilator and hardness using a Monsanto type hardness tester.

### *In Vitro* dissolution studies:

*In vitro* dissolution study of tablets performed using USP 24 type II dissolution apparatus (37±0.5°, 900 ml, 100 rpm) in phosphate buffer pH 7.2 for a period of 24 h. Aliquots were taken out at 0.5, 1.0, 1.5 and 2 h, and thereafter every hour for 24 h and the volume was replaced with an equivalent amount of aliquots of fresh dissolution medium. The samples withdrawn were analyzed. *In vitro* release of formulation was calculated using PCP Disso software (PCPD V 208). The computed values of kinetic constant (k) and diffusional release exponent (n) were determined.

### Optimization data analysis:

Various RSM computations for the current optimization study using RSM were carried out, employing software, State Ease Design Expert Version 7. Polynomial model including interaction and polynomial terms were generated for all the response variables. To investigate the influence of polymers on release pattern, a two way analysis of variance (ANOVA) based factorial analysis followed by several one way ANOVA. The general form of the model is represented as in Eqn. 1, Y=β_0_+β_1_X_1_+β_2_X_2_+β_3_X_1_X_2_+β_4_X^2^_1_+β_5_X_2_^2^+β_6_X_1_X_2_^2^+β_7_X^2^_1_X_2_+β_8_X_21_X_22_--(1), where β_0_ the intercept, is the arithmetic average of all quantitative outcomes of nine runs, β_1_ to β_8_ are the coefficients from the observed experimental values of Y. While X_1_ and X_2_ are the coded levels of the independent variable(s). The terms X_1_ X_2_ and X^2^_i_ (i=1, 2) are the interaction and polynomial terms, respectively. The stastical validity of the polynomials was established on the basis of Yates ANOVA. Subsequently, feasibility as well a grid search was performed to locate the composition of optimum formulations[14–18]. Also, three dimensional response surface graphs and contour plots were drawn in MS-Excel using the output files generated by the State Ease Design Expert Version-7 software.

Five optimum formulations were selected by intensive search, performed over the entire experimental domain, to validate the chosen experimental design and polynomial equations. The criterion for selection of optimum was primarily based on the desired values of the response parameters, i.e. release at 2 h and release at 18 h. The formulations corresponding to this optimum were prepared and evaluated for various response properties. Resultant experimental data was quantitatively compared with predicted values and linear regression plots was obtained using MS-Excel, forcing the line through origin.

## RESULTS AND DISCUSSION

**Fig. 1:**
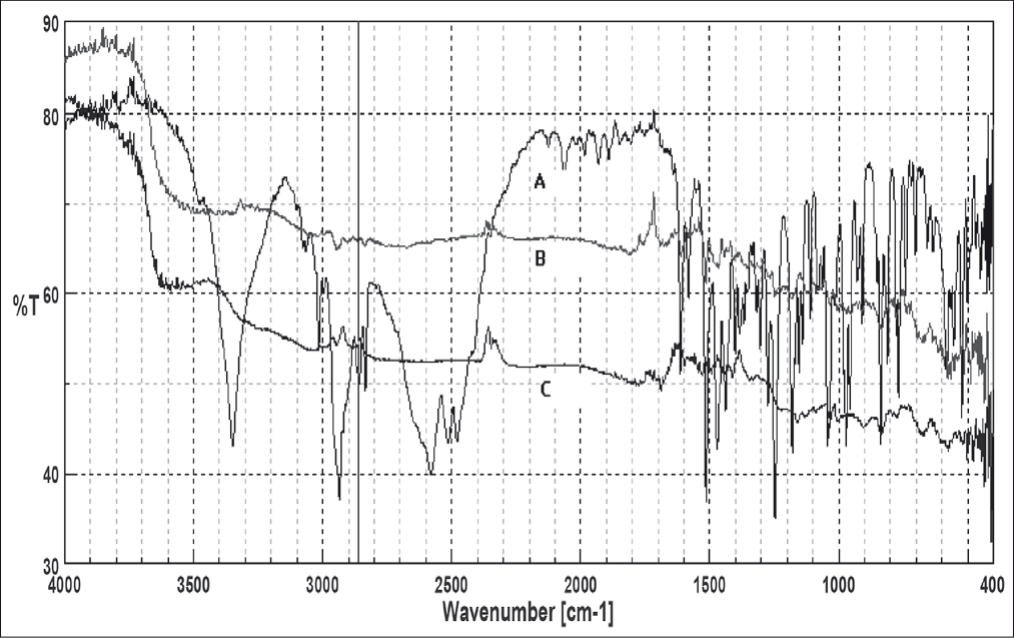
Infrared Spectra for complexation studies (A) venlafaxine HCl, (B) Drug resin complex and (C) indion 244

Time to reach ion exchange equilibrium was found to be 4 h. The IR showed in fig. 1 confirms the complex formation between drug and resin. The peaks representing amino group of the drug (3349 cm^−1^) and the peak at 2900 cm^−1^ corresponding to –OH stretching in drug are absent in DRC, which signifies that during DRC formation there was interaction of the amino group of drug with the sulfonic group of resin.

**Fig. 2:**
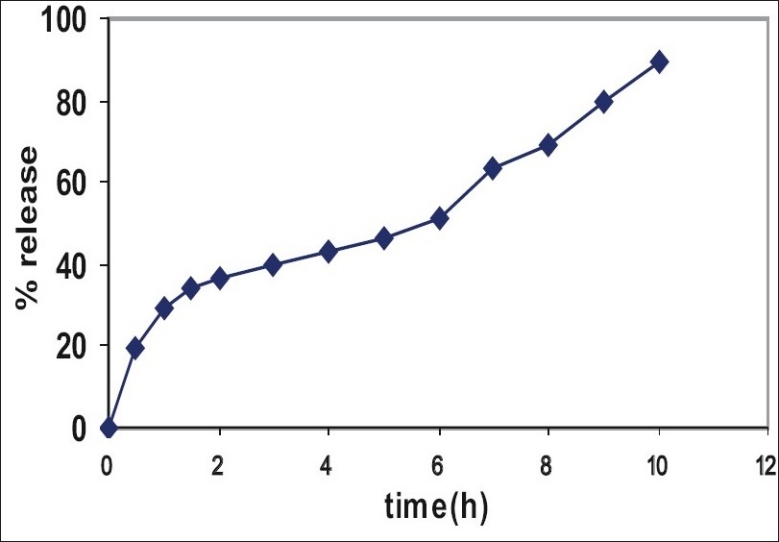
*In vitro* drug release from resinate.

*In vitro* dissolution from DRC showed inadequately sustained release (fig. 2). Sustained release pattern occurs due to the drug resin complex. In the dissolution medium the drug was then replaced by the counter ions of the dissolution medium. DRC showed inadequately sustained release. Hence to further prolong the release it was decided after preliminary studies to granulate resinate with ethyl cellulose and incorporation into HPMC matrix.

**Fig. 3:**
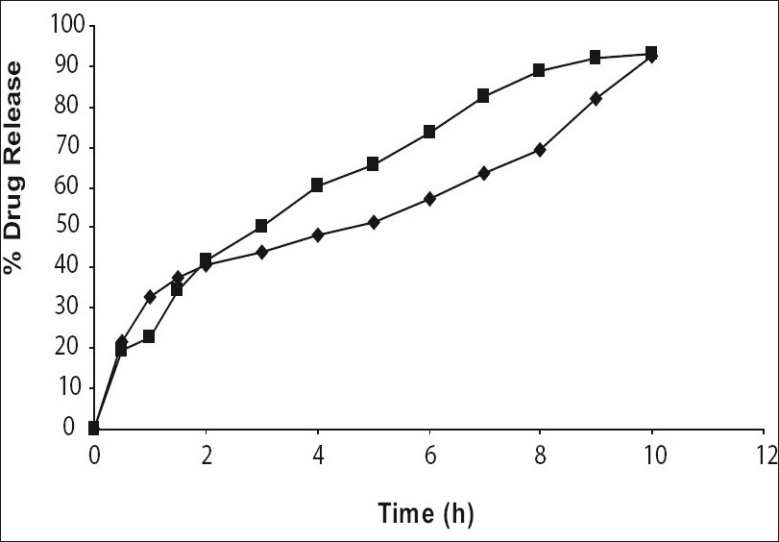
Effect of pH of dissolution medium on drug release through DRC DRC is drug resin complex. 0.1 N HCl (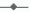); 7.2 pH PBS (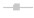).

Studies carried out to establish effect of pH of dissolution medium onto drug release through the DRC showed no significant effect of pH on drug release through DRC (fig. 3). The assay content of venlafaxine HCl varied between 98.0 and 99.8%. Tablets weights varied between 448 and 452 mg, thickness between 4.5 to 6.5 mm, and hardness between 8.0 and 9.0 kg/cm^2^ and friability ranged between 0.3 and 0.6%.

Table 3 lists various dissolution kinetics parameters computed for all nine batches. In our experiments, the *in vitro* release profiles of the drug from all the formulations could be best expressed by Peppas Eqn. When plotted according to Korsmeyer's Eqn, formulations also showed high linearity (R^2^: 0.9985), with a comparatively high slope (*n*) value of ranging from 0.4381 to 0.9119. This *n* value, however, appears to indicate a coupling of diffusion and erosion mechanisms so called anomalous diffusion. The relative complexity of this formulation and its components may indicate that the drug release is controlled by more than one process. The values of *n* show increasing trend with increase in HPMC content, even at higher EC levels.

**TABLE 3:** DISSOLUTION PARAMETERS FOR ALL SUSTAINED RELEASE FORMULATION PREPARED AS PER 3^2^ FACTORIAL DESIGN

Trial no	HPMC (mg)	EC (mg)	K	*n*	Release 2 h	Release 18 h	r^2^
F_1_	41.62	41.62	26.84	0.4381	38.97	95.35	0.9066
F_2_	41.62	83.24	15.27	0.6250	30.97	89.93	0.9499
F_3_	41.62	124.86	15.12	0.5098	33.47	87.69	0.9678
F_4_	83.24	41.62	5.50	0.9119	13.21	86.54	0.9700
F_5_	83.24	83.24	6.67	0.7820	14.30	83.18	0.9882
F_6_	83.24	124.86	7.78	0.7734	16.33	88.51	0.9883
F_7_	124.86	41.62	5.71	0.8653	11.99	82.55	0.9893
F_8_	124.86	83.24	8.53	0.7328	16.57	80.85	0.9766
F_9_	124.86	124.86	8.53	0.7328	18.09	82.22	0.9743

F_1_-F_9_=Nine formulations designed by factorial designs, HPMC= hydroxypropylmethylcellulose, EC= ethylcellulose, K= kinetic constant, *n*= diffusion coefficient, r^2^= coefficient of determination.

Further, value of kinetic constant (k) ranged between 5.50 and 26.84. The values of release after 2 h varied between 11.99 and 38.97, and release after 18 h found between 80.85 and 95.35. HPMC K 15 M was used as a hydrophilic matrimixing agent because it forms a strong viscous gel on contact with aqueous media, which could be useful in controlling delivery of highly water soluble drugs[19]. HPMC tablets were able to sustain the drug release alone but could not control the initial release i.e. release is more than 20% in the initial 2 h. Faster release of drug from the hydrophilic matrix was probably due to the faster dissolution of the highly water soluble drugs from the core and its diffusion out of the matrix forming the pores for entry of solvent molecules. Incorporation of ethylcellulose was found to control the drug release to some extent, which could be attributed to the decreased penetration of the solvent molecules in the presence of hydrophobic polymer leading to decreased diffusion of the drug from the matrix.

The drug release from cellulose ether matrix tablets can be controlled by the type of polymer and its molecular weight[19]. When EC and HPMC are combined, the hydrophobic interaction of EC retarded the water uptake while HPMC contributed by absorbing water to swell. As a result tablet swells initially and then erode, so that initial release is controlled[20].

**Fig. 4:**
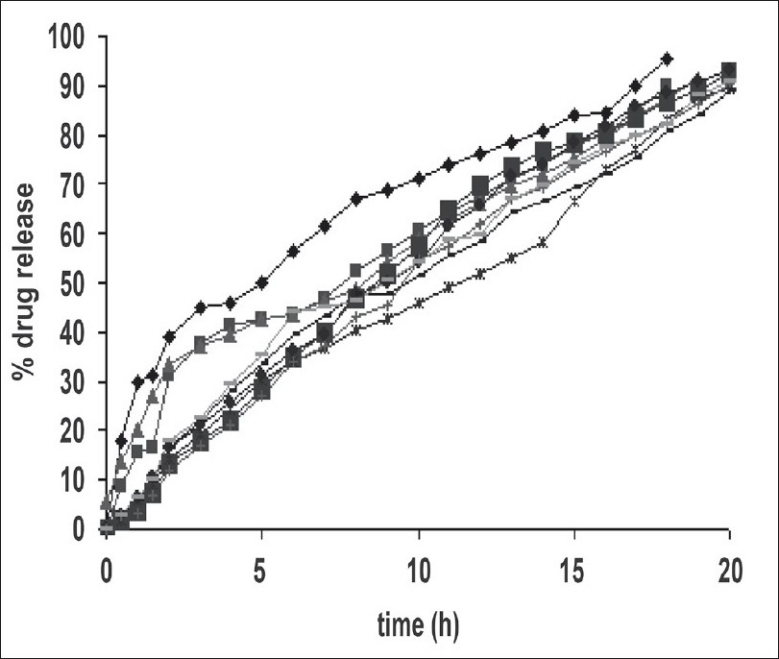
Comparative *in vitro* dissolution profile of sustained release formulation of venlafaxine as per 3² factorial design F_1_ (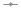); F_2_ (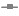); F_3_ (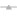); F_4_ (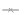); F_5_ (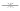); F_6_ (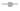); F_7_ (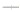); F_8_ (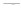); F_9_ (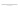)

At lowest concentrations of the HPMC and EC, compressed matrices showed increased drug release in the initial 2 h as shown in fig. 4. This is due to decrease in hydrated gel layer formation, but at the high concentration of HPMC and EC initially tablet shows increased in the diffusion layer formation probably due to the increased concentration of HPMC followed by erosion of tablet was observed.

An increase in the amount of polymer will decrease the drug release. Sustained drug release was seen with the highest levels of the two polymers. Application of two-way ANOVA based factorial analysis indicated that the polymers had a significant influence on the initial release of drug within 2 h from the compressed matrices (*P*<0.05). Subsequent application of one-way ANOVA, keeping the levels of one of the polymers fixed, also showed a statastically significant difference amongst the observed data of dissolution (*P*<0.05), ratifying the significant positive influence of each polymer on dissolution.

The mathematical relationships constructed for the studied response variables are expressed as Eqns. 2 and 3. All the polynomial equations were found to be statistically significant (*P*<0.05), as determined by ANOVA;

Release at 2 h= 22.84-9.16X_1_+3.40X_2_-0.42Y_1_+0.11Y_2_+4.83 X_1_Y_1_-1.06 X_2_Y_1_-0.64 X_1_Y_2_-0.034 X_2_Y_2_--(2), Release at 18 h= 87.49-3.33X_1_-0.45X_2_-1.35X_2_Y_1_+0.43Y_2_+2.11X_1_Y_1_-1.25X_2_Y_1_+ 0.31X_1_Y_2_-0.37X_2_Y_2_--(3)

The polynomial equations comprise the coefficients for intercept, first-order main effects, interaction terms, and higher-order effects. The sign and magnitude of the main effects signify the relative influence of each factor on the response. At a given set of factor levels, however, these higher-order polynomials yield results as the net effect of all the coefficient terms contained in the polynomial.

**Fig. 5:**
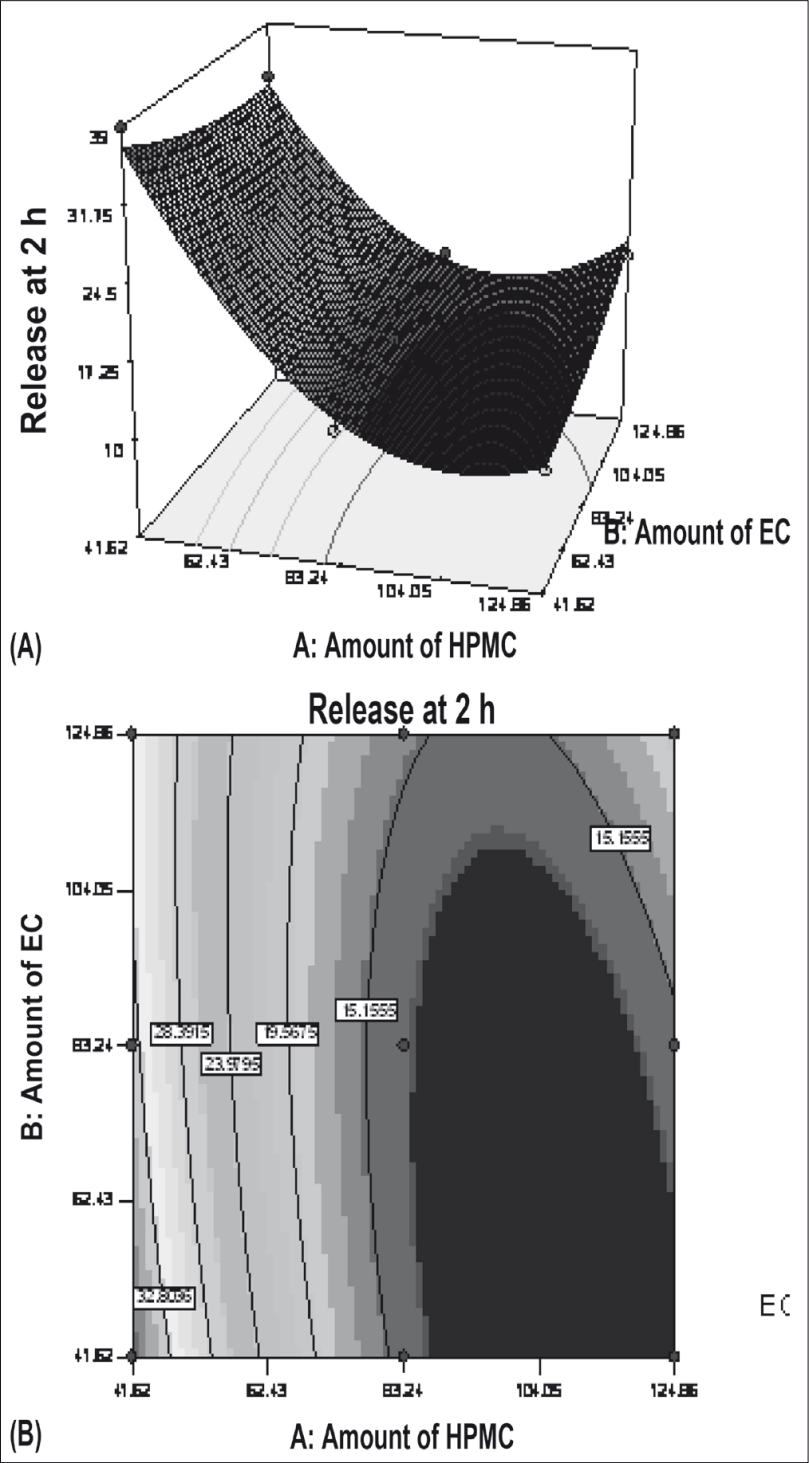
Influence of HPMC and EC on the release at 2 h. HPMC is hydroxypropylmethylcellulose and EC is ethylcellulose. A) Response surface and B) contour plot; X1= A:Amount of HPMC, X2= B:Amount of EC

**Fig. 6:**
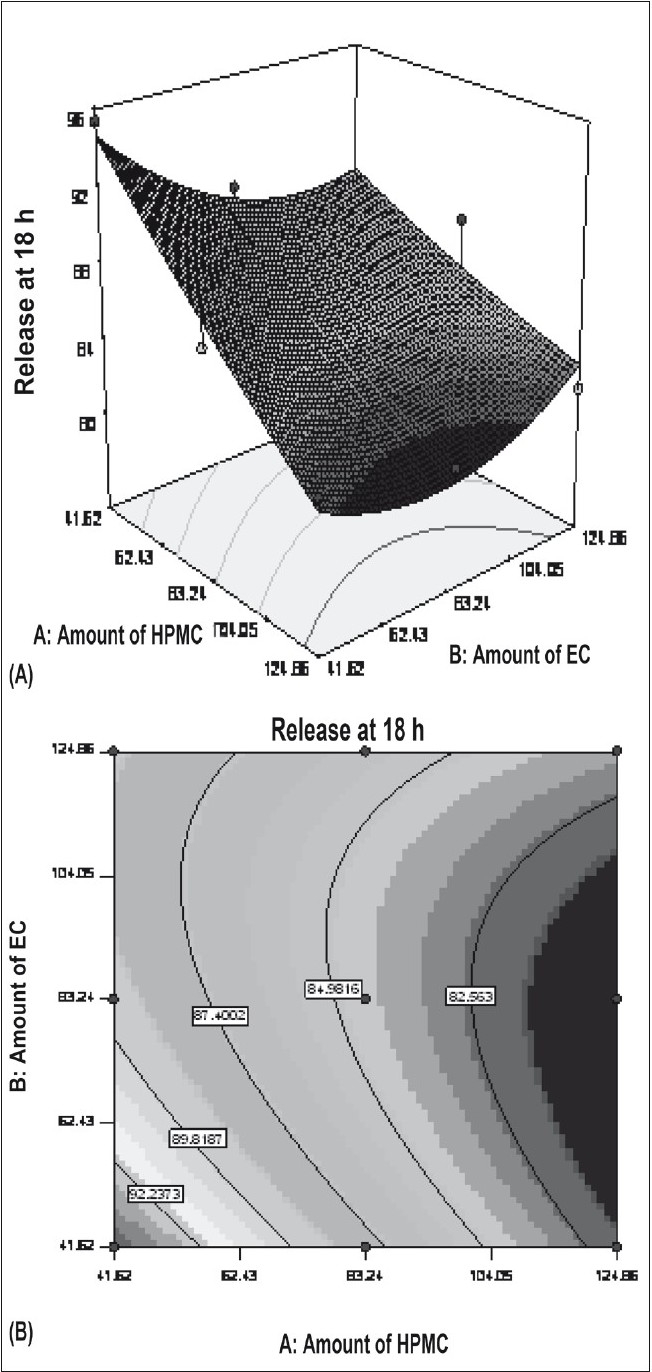
Influence of HPMC and EC on the release at 18 h HPMC is hydroxypropylmethylcellulose and EC is ethylcellulose. A) Response surface and B) contour plot; X1= A:Amount of HPMC, X2= B:Amount of EC

Figs. 5 and 6 portray the 3 dimensional response surface plots and the corresponding contour plots for the studied response properties viz rel_2h_ and rel_18h_, respectively. Fig. 5, shows that release after 2 h varies in a nearly linear descending pattern with a change in the amount of polymers. Fig. 6 also exhibits a near linear trend of release after 18 h, but in ascending order. As there is no confounding of the contour lines in figs. 5 or 6, both the polymers seem to contribute independently towards drug release. For all of the 5 checkpoint formulations, the results of the dissolution parameters showed in Table 4. Table 5 lists the compositions of the checkpoints, their predicted and experimental values of all the response variables, and the percentage error in prognosis.

**TABLE 4:** DISSOLUTION PARAMETERS OF FIVE OPTIMUM FORMULATIONS

Formulation Code	HPMC (mg)	EC (mg)	K	*n*	r^2^	Release 2 h	Release 18 h
O_1_	99.88	41.62	8.7519	0.8109	0.9972	18.02	86.92
O_2_	101.96	46.81	8.0564	0.8314	0.9974	17.94	86.55
O_3_	105.08	47.86	8.5724	0.8229	0.9973	16.32	86.23
O_4_	106.13	49.94	8.1089	0.8327	0.9978	16.88	85.98
O_5_	108.21	45.78	6.9683	0.8719	0.9985	16.26	85.64

O_1_-O_5_= Codes for optimized formulations, HPMC= hydroxypropylmethylcellulose, EC= ethylcellulose, K= kinetic constant, *n*= diffusion coefficient, r^2^= coefficient of determination.

**TABLE 5:** EXPERIMENTALLY OBSERVED RESPONSE PARAMETERS OF FIVE OPTIMUM FORMULATION AND COMPARISON WITH PREDICTED VALUES FOR VALIDATION OF RSM

Formulation Code	Formulation Composition HPMC/EC (mg)	Response Property	Experimental Value	Predicted Value	Percentage Error
O_1_	99.88/41.62	% Release 2 h	18.02	18.22	-1.560
		% Release 18 h	86.92	87.18	-0.299
O_2_	101.96/46.81	% Release 2 h	17.94	17.91	0.610
		% Release 18 h	86.55	86.81	1.151
O_3_	105.08/47.86	% Release 2 h	16.32	16.96	-3.921
		% Release 18 h	86.23	86.59	-0.417
O_4_	106.13/49.94	% Release 2 h	16.88	17.07	-4.852
		% Release 18 h	85.98	86.34	-0.453
O_5_	108.21/45.78	% Release 2 h	16.26	16.44	-1.107
		% Release 18 h	85.64	86.22	-0.677
Mean (±SD) of % error					-1.152±1.88

O_1_-O_5_= Codes for optimized formulations, HPMC= hydroxypropylmethylcellulose, EC= ethylcellulose.

**Fig. 7:**
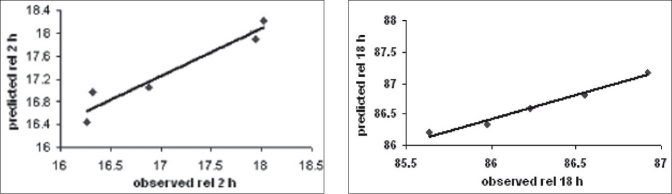
Linear correlation plots between observed and predicted values Linear correlation plots between observed and predicted values for (A) release at 2 h and (B) release at 18h.

Fig. 7 showed linear correlation plots between the observed and predicted response variables. The predicted error for the response variables ranged between -4.852% and 1.151%, with the mean±standard deviation of the percentage error being −1.152±1.88%. Also, the linear plots drawn between the predicted and observed responses for release at 2 h and release at 18 h demonstrated high of r^2^ 0.9296 and 0.9765, respectively (fig. 7), indicating excellent goodness of fit. Thus, the low magnitudes of error, as well as the significant values of r^2^, designate a high prognostic ability of RSM.

Regulated drug release study indicates that the hydrophilic and hydrophobic matrix tablets of venlafaxine, prepared using HPMC and EC, can successfully be employed as a once-a-day oral controlled release drug delivery system. Both the polymers could extent the drug release upto 20 h so it helps to achieve the low plasma concentration to reduce side effects. However, appropriate balancing between various levels of the 2 polymers is imperative to acquire proper sustained release. High degree of prognosis obtained using RSM corroborates that a 2-factor CCD is quite efficient in optimizing drug delivery systems that exhibit nonlinearity in response(s).
